# The current state of wearable device use in Parkinson's disease: a survey of individuals with Parkinson's

**DOI:** 10.3389/fdgth.2024.1472691

**Published:** 2024-12-23

**Authors:** Siegfried Hirczy, Cyrus Zabetian, Yi-Han Lin

**Affiliations:** ^1^ Department of Neurology, Veterans Affairs Puget Sound Health Care System, Seattle, WA, United States; ^2^ Department of Neurology, University of Washington, Seattle, WA, United States

**Keywords:** Parkinson's disease, wearable, clinical practice, survey, barriers to use, consumer devices, medical devices

## Abstract

**Background:**

Interest in wearable device use in Parkinson's disease (PD) has grown rapidly with many compelling studies supporting diagnostic and therapeutic uses. Concurrently, consumer devices have proliferated and their role in health and wellness has expanded. However, incorporation of consumer and medical wearable devices into medical care has in our experience been limited.

**Objective:**

We sought to assess the current state of consumer and medical wearable device use among those with PD and to understand the factors impacting their rate of use.

**Methods:**

An anonymous online survey of individuals with PD in the US was conducted from July 9th, 2023, to Jan 8th, 2024, with 298 completed responses collected.

**Results:**

Greater than 90% of respondents were interested in new technologies with 67% having had experiences with consumer wearable devices. Only 24% were using consumer devices for disease management and many functions were not fully utilized. Medical wearable device use was very limited with only 8% having used a device. Users of both consumer and medical wearables generally reported low barriers to use despite continued strong perceptions on the importance of cost, impact on care, comfort, and other factors.

**Conclusion:**

This study demonstrates that for the clinical management of PD there is limited use of wearable devices even among individuals who are motivated and experienced with consumer wearable device use. Additionally, it is suggested that substantial barriers to medical wearable use are likely originating from the provider and/or systemic level.

## Introduction

Parkinson's disease (PD) is a neurodegenerative condition which results in abnormal movements, cognitive changes, and autonomic dysfunction. Diagnosis and management are made challenging due to inherent fluctuations in the severity of disease manifestations as well as dynamic changes that are induced by treatment. Standard clinical practice can only capture brief snapshots of the patient's experience and relies heavily on subjective report and quasi-objective exams, thus ability to optimally intervene is limited (1–4). Recently, there has been considerable interest in addressing these challenges through the creation of objective and continuous measures which hope to allow for better understanding of each person's unique disease state and thereby improve treatment and reduce disability (5).

Wearable devices have been of great interest in healthcare due to their ability to contain imbedded sensors that help track various physiologic signals. Research in wearables in Parkinson's disease has been focused on the evaluation of motor features, and various devices have data supporting their ability to detect sub-clinical motor features, disease state fluctuations, disease progression, and to assist with therapeutic adjustments (6–9). However, various physiologic signals such as sleep (10), heart rate variability (11), cerebral oxygenation (12), and many others have been studied in PD with wearables. Studies in this area have rapidly increased over time with PubMed entries for “wearable” AND “Parkinson's Disease” going from a mere seven in 2012 to 170 in 2022. To help researchers and clinicians understand this landscape, many excellent reviews are available (13–18).

However, collection of valid data alone is not sufficient to change practice. The patient's perspective on device usability and utility is critical, and this has not been forgotten. Many studies of specific devices have included patient perspectives on features of interest, usability, and barriers to use (19–21). Additionally, more conceptually focused studies using surveys and focused groups have identified key features of interest such as wearability, ability to provide feedback, and clinical accuracy (22, 23). All this research has ultimately culminated in several medical wearable devices that are validated, designed with the patient in mind, and cleared for clinical use.

Concurrent with this explosion of research and approval of medical wearable use, there has been substantial adoption of consumer wearable devices for health tracking and lifestyle management. Devices such as the Apple Watch and Fitbit were in 2020 estimated to be used by around 25%–30% of the US population for health monitoring (24). These consumer devices also appear to have merits in PD as they offer the ability to potentially improve medication adherence, encourage and track physical activity, document symptoms, monitor sleep, and collect various other forms of information.

However, despite the extensive research on validation, the understanding of the factors important to patient users available to device manufacturers, the widespread use of consumer wearable devices, and the availability of approved medical devices for clinical use, real-world clinical data appears to be scant. In our clinical experience and after discussion with colleagues, few individuals are using these devices. While issues with the payor model for device use, lack of clinical impact, poor tolerability for patient and clinician users, and general disinterest in new technologies are commonly mentioned as factors playing a role, the evaluation of these barriers has not been extensively evaluated in routine clinical care.

We therefore sought to conduct a comprehensive evaluation of the current usage of wearable devices in PD, and to go beyond the controlled research setting to understand the real-world usage of wearable devices both consumer and medical. Additionally, we wanted to understand what factors were currently playing a role in current device usage and whether these were the same as those reported previously.

## Methods

An anonymous online survey was conducted from July 9th, 2023, to Jan 8th, 2024. Respondents were self-identified individuals with PD and were requested to be at least 18 years of age.

Wearables were defined in this survey as any technological accessory which is affixed to the surface of an individual and which provides information on their movements (monitoring devices). A medical wearable device definition was not supplied, but options were explicitly listed (Apple Watch with StrivePD, PKG, KinesiaU, PDMonitor). Of note, given that StrivePD is an application that functions on a consumer device, we asked users of StrivePD to answer both as consumer wearable device Apple Watch users and as medical wearable device users.

The survey was designed by the study team with input from other specialist clinicians. Question topics included basic demographics, disease state, understanding and use of wearable devices (divided into consumer and medical device categories), general perceptions as related to theoretical devices, and general barriers to use. Survey questions were generally multiple-choice questions, but free response sections were provided in many cases to allow participants to provide answers that were not accounted for by the survey developers (Supplementary Survey Document).

Recruitment was conducted by collaborating groups who distributed study-related information and a link to the survey. The American Parkinson Disease Association (APDA) and the Washington State Parkinson Disease Registry participated in participant outreach (25).

Interested individuals followed the link and were brought to the online REDCap electronic data capture tool. The first page of the survey provided potential participants with information about the study and associated risks and benefits. Interested individuals would electronically confirm that they consented to participate, which would then allow them to proceed to the survey content (26, 27).

After survey completion, PD disease status nor any other characteristics of participants were verified. It was determined that verification would have limited the response rate and would have introduced more risk of identification and more bias as the systems available for use would tie individuals to specific medical systems. Additionally, it was believed that false representation was unlikely, as the survey was targeted, a response required substantial effort, and no notable financial incentive was present for respondents.

After survey closure, data processing and statistical analysis were performed using the R statistical analysis platform. Targeted sub-group analysis evaluating the effects of demographic and disease features on perceptions and experiences was performed post-hoc.

## Results

### Survey response, demographics, and disease state

A total of 346 responses were collected with 298 completed surveys (86%). The response rate was unknown but was suspected to be very low given the size of the APDA distribution network. Only completed surveys were included in the analysis. Responses came from individuals living in 28 states with the greatest number coming from Washington State (63%) (Supplementary Table S1). A limited set of demographic features were recorded (Table 1). Disease related symptoms and characteristics varied encompassing both early and late stages of disease (Supplementary Tables S2, S3).

**Table 1:** Respondent demographic characteristics (*n* = 298).

Characteristics	Count	Percentage
Age		
<40	1	0.3%
40–50	9	3.0%
50–60	38	12.8%
60–70	110	36.9%
70–80	120	40.3%
80+	20	6.7%
Gender		
Male	128	43.0%
Female	169	56.7%
Prefer not to answer	1	0.3%
Residential setting		
Suburban	155	52.0%
Urban	92	30.9%
Rural	51	17.1%
Care setting		
Private/Non-university	180	60.4%
Tertiary/Academic	77	25.8%
VA/National Gov.	25	8.4%
County/Public	16	5.4%

### Technology and consumer device experiences

**Figure 1:**
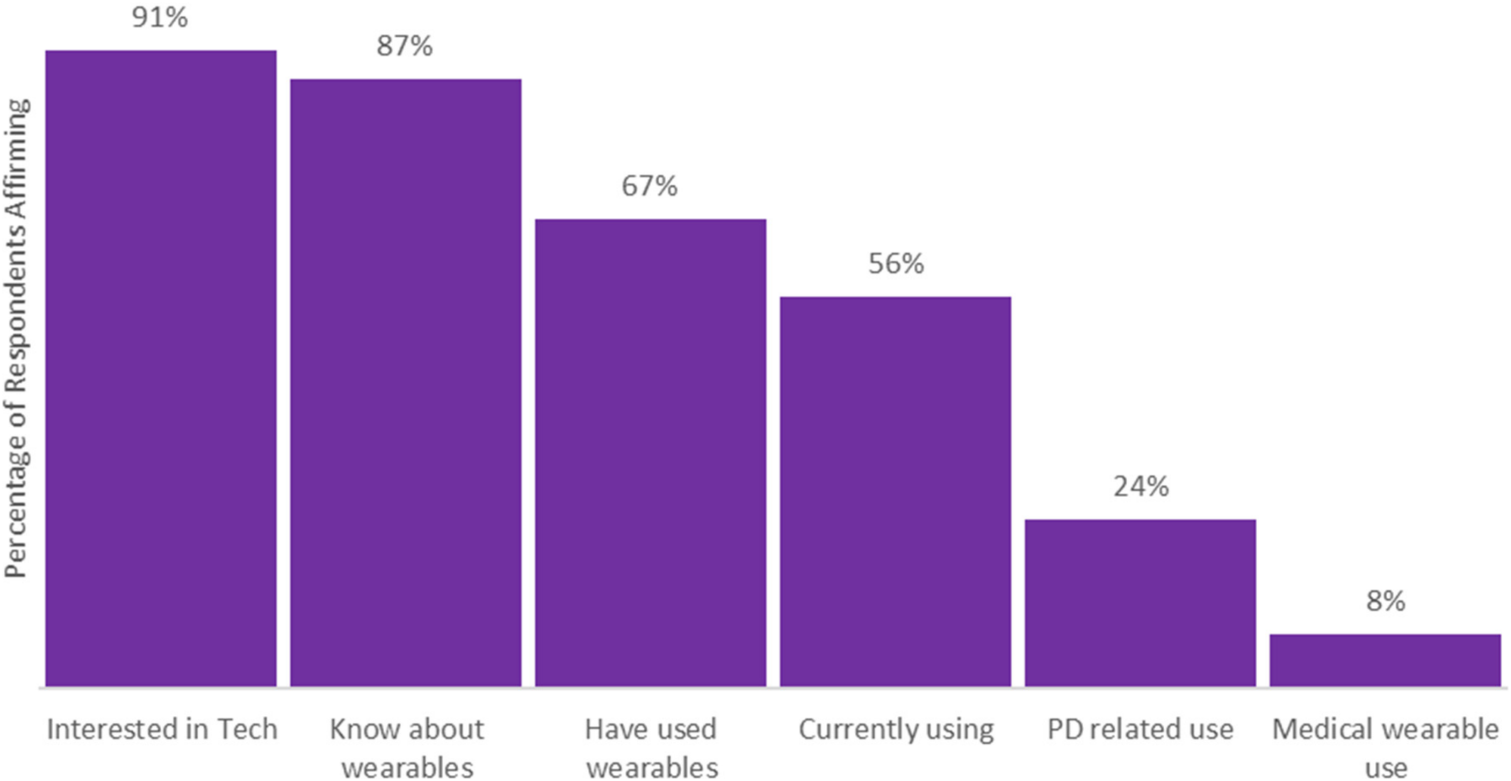
Respondent interest, knowledge, or experiences with wearable device use. For the interested in technologies category answers of moderately or very interested were combined, neutral or below were not incorporated. All other questions were yes or no responses.

Regarding technology and wearable device use, there was a high degree of interest in new technologies with 91% of individuals either very or somewhat interested. Knowledge about and use of wearable devices was also high with 87% knowing about wearable devices, 67% having used a device, and 56% currently using one (Figure 1). Most respondents knew about smart watches and fitness trackers (Supplementary Table S4); the Apple Watch was the most used and most preferred consumer device among respondents (Supplementary Table S5). Device use retention rates were also high with 84% of those with experience with wearable devices continuing to use a device. Among all device users, device usage time was very high with 90% using their preferred device nearly always or at least all the time while awake (Supplementary Table S6).

### Consumer wearable device feature use

**Figure 2:**
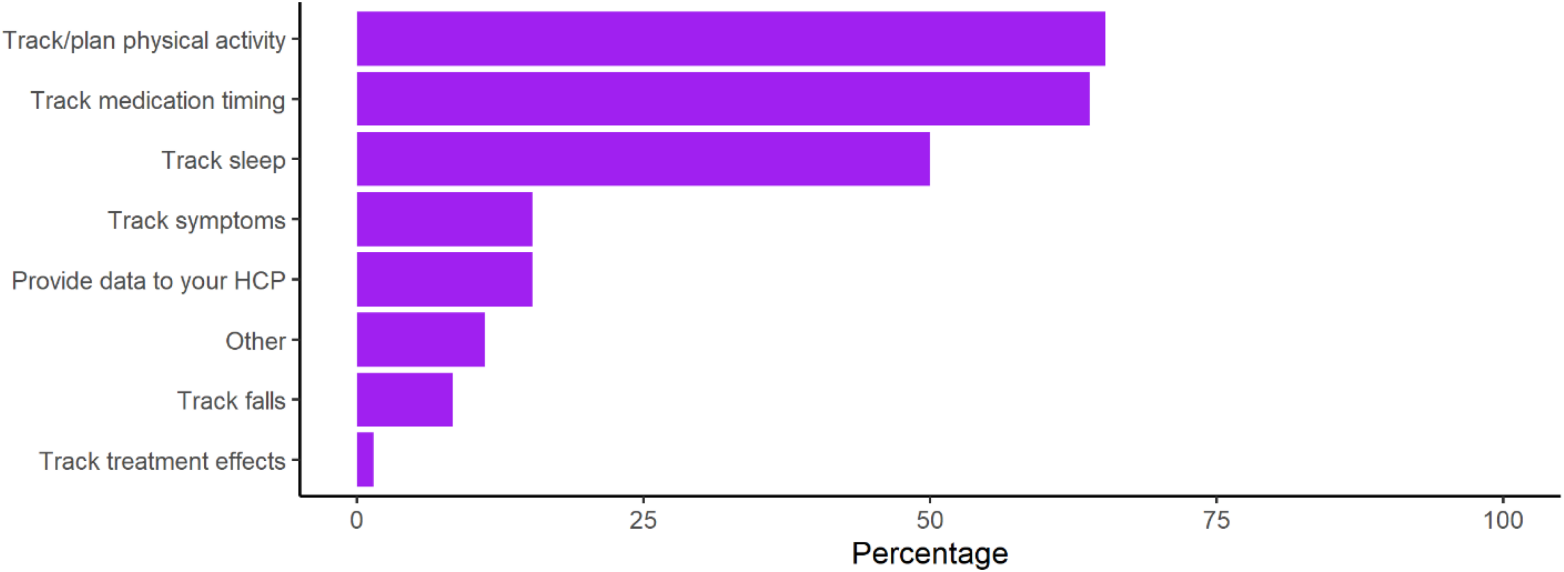
Use of specific consumer device functions for the management of PD. Calculated as the percentage of respondents using each function from the subset of respondents who affirmed that they were using consumer wearables to manage PD (*n* = 72).

However, use of wearable devices for the management of PD was low at only 24% of respondents. Among those using wearable devices, individuals were using them most to track physical activity, medication timings, and sleep (Figure 2). Post-hoc subgroup analysis of Apple Watch wearable device users was performed to evaluate whether device capability limitations were playing a large role in lack of management related use, however findings mirrored those seen among all device users (Supplementary Figure S1).

### Consumer wearable devices effect on disease management

Among those using wearables to manage PD, 76% reported a positive impact on personal management of PD (38% substantially, 39% somewhat), 24% reported that use was not particularly impactful, and no negative responses were recorded. Impact on medical team management of PD was felt to be not particularly impactful by 57% of respondents, though substantially positive (10%) or somewhat positive (33%) responses were reported, and no negative responses were reported.

### Consumer wearable device barrier to use

Among survey respondents, current device use was limited most frequently because of lack of knowledge about the abilities of wearable devices and by cost (Supplementary Table S7). More generally, individuals were also surveyed on the use of smartphones and other applications for PD monitoring with 26% reporting the use of an application.

### Medical wearable device usage and barrier to use

Medical wearable device use among respondents was 8% (*n* = 23). There were 19 StrivePD, 3 PD Monitor, 1 PKG, and 0 Kinesia users. Device use frequency was variable and many limitations on use were noted (Table 2). Impact on personal management was 26% positive (9% substantially, 17% somewhat) and impact on medical team management was 30% positive (4% substantially, 26% somewhat) (Supplementary Table S8).

**Table 2:** Respondent medical wearable device use, frequency of use, and limitations on use. Other limitations reported for Strive PD were that the device was in the process of being setup (1), difficulties logging in (1), geographic limitations on use (1), and an allergic response (1).

	Device			
	Any	StrivePD	PDMonitor	PKG
Total users	23	19	3	1
Use frequency				
Nearly always	43%	47%	33%	0%
All day when awake	26%	32%	0%	0%
>50% of wake time	4%	0%	33%	0%
Not daily but more than 3 days a week	4%	5%	0%	0%
Weekly	0%	0%	0%	0%
Monthly	0%	0%	0%	0%
Less than monthly	0%	0%	0%	0%
No longer using	22%	16%	33%	100%
Limitations on use				
No limitations	43%	47%	33%	0%
Uncomfortable/Difficult to keep on	0%	0%	0%	0%
Too much effort to maintain	9%	11%	0%	0%
Insufficient capabilities	9%	11%	0%	0%
Data input difficulties	17%	16%	0%	100%
Data review or access difficulties	4%	5%	0%	0%
Lack of impact on PD	26%	21%	67%	0%
Not utilized by healthcare provider	13%	11%	33%	0%
Concerns about accuracy of data	4%	5%	0%	0%
Discontinued by healthcare provider	4%	0%	33%	0%
Cost issues	0%	0%	0%	0%
Other	17%	21%	0%	0%

### General wearable interest and limitations

To separate perceptions and experiences tied to current consumer or medical devices, we asked about two similar theoretical devices. We described a version which provided information to the patient but did not directly provide it to the healthcare team (Type A) and one that provided information directly to both (Type B). For both versions, individuals were interested in using such a device (Supplementary Figure S2). Additionally, among those who would consider using the devices greater than 90% of respondents were willing to use either device at least all day while awake (Supplementary Figure S3). However, only 49% of those interested were willing to pay for a device if not covered by insurance. Of those who were willing to pay for such a device, the median one-time payment was $200 for both and the mean $252 (Type A) and $259 (Type B). Alternatively, we also asked about subscription pricing and what individuals would be willing to pay with the median being $10 for both devices and mean being $14 (Type A) and $15 dollars (Type B). There was no difference between the cost individuals were willing to pay for device [*p* = 0.6 (lump sum) and *p* = 0.2 (subscription), Wilcoxon paired signed rank test in the setting of non-normality of data shown by Shapiro-Wilk testing].

**Figure 3:**
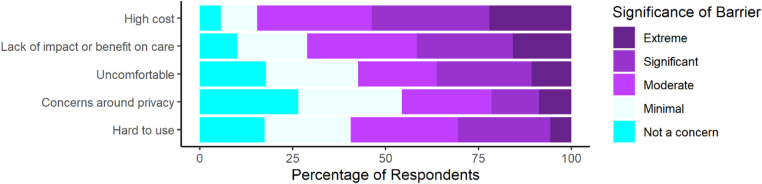
Perceived significance of certain barriers to respondents’ personal use of wearable devices for management of PD as rated on a 5-point scale from not a concern to an extremely significant barrier (*n* = 298).

Finally, individuals were surveyed on barriers to their use of wearable devices for PD (Figure 3). Respondents reported concerns about all surveyed barriers with most respondents reporting at least a moderate level of concern for 4 out of the 5 surveyed barriers. Respondents reported the largest proportion of extreme concern for cost and impact on care. Additionally, we compared perceptions between individuals with different levels of wearable device experience (Supplementary Figure S4).

## Discussion

This study captured wearable device experiences and perceptions among individuals with PD. Respondents were very technologically inclined (91% reporting interest) and were more likely to use wearable devices than the general US population, 67% vs. 35% (Morning Consult, Survey, 2023). Consumer devices among respondents also appeared to be well tolerated as discontinuation of wearables was rare and users also wore the devices most of the time.

However, despite the barriers to general consumer use being overcome, consumer wearable use for the specific purpose of managing PD was strikingly low at 24% and use of commonly available devices functions was low. This incomplete pattern of usage remained true even after removing ambiguity in the definition of “management” and ensuring that all functions queried were possible. In this subset, despite previous reported interest (28) and the importance placed on these features in PD, less than three quarters reported using the device to track physical activity, less than half tracked sleep, and less than a quarter tracked symptoms. Participants reported the lack of PD related use to be most often due to knowledge of functionality (26.2%). Features previously noted as important to address such as difficulties with wearability or comfort (4.7%), data input (9.4%), and data review (5.0%) were not prominently reported (23). These findings are supportive of the acceptability and usability of current consumer devices in PD.

While consumer wearable device use for PD was sub-optimal, medical wearable device use was marginal. Only 8% of respondents had any experience with them, which was less than 1/8th the number of consumer wearable device users. However, there did not appear to be marked barriers to use once implemented, given 43% of respondents reported no limitations. The most noted barrier to use was lack of impact on care 26% and this was additionally supported by most medical device users indicating that their devices had negligible impact on their healthcare providers' management of PD and even on their own management. However, other issues appear to be reasonably addressed with less than 20% reporting difficulties with data input, less than 10% issues with wearability, and less than 5% issues with data review.

To better understand what factors were limiting wearable use and to compare current perceptions to prior research, many questions were directed to assess their perceptions on the significance of certain barriers, and it was again seen that individuals with PD were concerned about comfort, usability, and impact on care, as well as cost and privacy (23). However, these results seemingly conflict with the results obtained from direct questioning about their personal wearable use. Due to this conflict we sought to assess whether this was due to differences between users with more and less experience. However, consumer and medical wearable users still appeared to endorse similar levels of concern. These findings therefore suggest that while certain factors are still of high importance to people with PD they have generally been addressed by the current generation of devices.

Ultimately, it appears that the barriers to medical wearable device use and to a lesser degree consumer wearable device use in Parkinson's disease do not stem from individuals with PD. Even when an individual with PD is motivated, experienced with wearable use, and interested in theoretical medical wearable devices there is still a high likelihood that they will not be a wearable device user. We believe that this reflects difficulties with technology and device integration at the level of the provider user and/or the healthcare system.

The main strengths of this study were the number of respondents, the diversity of disease stages included, the inclusion of multiple wearables, and the level of detail obtained about perceptions and experiences. As with most survey studies, there were meaningful limitations. Foremost among them was sampling bias which was likely substantial given the online format and low estimated respondent rate. Respondents were likely highly motivated and technologically inclined. The use patterns and perceptions noted in this study do not directly reflect those of the population. However, they retain value as they are almost certainly a reflection of the upper bound of device use and their perceptions likely reflect the most positive reflections of the population, as such one can reasonably infer that the population rate of experience is lower and that perceptions are likely to be less positive than were seen in this sample. Additionally, the survey was heavily biased towards the Pacific Northwest region of the United States despite the goal of having a national distribution, demographic and socioeconomic data was limited, and the number of respondents for questions relating to consumer wearable device use in PD and medical wearables was relatively small.

Future studies should work to better understand the true perceptions of the PD population by expanding distribution, reducing respondent barriers, collecting more demographic and socioeconomic data, and engaging the community. Furthermore, future longitudinal studies should be performed to assess the evolution of individual perceptions of wearables as they evolve in relation to exposure to wearable devices and disease progression.

In conclusion, this study confirms the existence of a highly motivated subpopulation of individuals with PD who have a strong interest in wearable devices and confirms the feasibility of high levels of wearable device use in real-world use. Novelly, it identifies and partially quantifies large gaps in the use of consumer wearable device health tracking features and integration of wearable devices into PD related health management. Additionally, it confirms that medical wearable device use is low, but suggests that this isn't an issue with patient usability, thereby implicating provider and/or systemic barriers as the bottleneck to medical device use. We believe that these results call for further investigation into understanding the barriers affecting real-world use among clinician users and healthcare systems, as well as studies targeted at enhancing the utility and understanding of all forms of wearable device use in PD.

## Data Availability

The raw data supporting the conclusions of this article will be made available by the authors, without undue reservation.

## Supplementary material

Supplementary Figure S1 Use of specific consumer device functions for the management of PD. Calculated as the percentage of respondents using each function from the subset of respondents who affirmed that they were using consumer wearables to manage PD and who were using the Apple Watch as their preferred device (*n* = 41).

Supplementary Figure S2 Respondent degree of interest in using theoretical wearable devices in PD. Device A was defined as a device with the ability to help individuals better understand their disease and responses to medical interventions, but without ability to directly provide information to healthcare providers. Device B was similarly defined but with the added benefit of healthcare provider integration (*n* = 192).

Supplementary Figure S3 Amount of time respondents were willing to wear theoretical devices A and B if there were no issues with comfort or usability. (Device A *n* = 293, Device B *n* = 292).

Supplementary Figure S4 Significance of barriers to wearable device use among individuals with different degrees of experience with wearable device use. MWU = medical wearable device user, CWU + PD = consumer wearable device user who manages PD with the device. CWU-PD = consumer wearable device user not managing PD with the device. Ex CWU = participants who have used consumer wearable devices but no longer are. Non-User = no experience with wearable devices (*n* = 298).

Supplementary Table S1 States in which survey respondents reside (*n* = 298).

Supplementary Table S2 Respondent PD related characteristics and symptom characteristics (*n* = 298).

Supplementary Table S3 Respondent assistive gait device utilization frequencies among those who reported using an assistive device (*n* = 58).

Supplementary Table S4 Respondent awareness of and experiences with wearable devices (*n* = 298).

Supplementary Table S5 Respondent wearable use preferences among those with experience with wearable devices (*n* = 193). Other responses for preferred device: apollo neuro (1), iphone (1), kospet (1), and Samsung watch (1).

Supplementary Table S6 Preferred consumer wearable device use time among individuals still using thei preferred device (*n* = 160).

Supplementary Table S7 Respondent reported current barriers to the use of consumer wearable devices for the management of PD. Individual respondents could affirm the presence of multiple barriers.

Supplementary Table S8 Medical wearable device user perceptions on impact of devices on management of PD (*n* = 23).
