# Supplementary figures and images for: The current state of wearable device use in Parkinson's disease: a survey of individuals with Parkinson's
Source: Front Digit Health. 2024 Dec 23;6:1472691. doi: 10.3389/fdgth.2024.1472691 (PMC11701158; doi:10.3389/fdgth.2024.1472691)

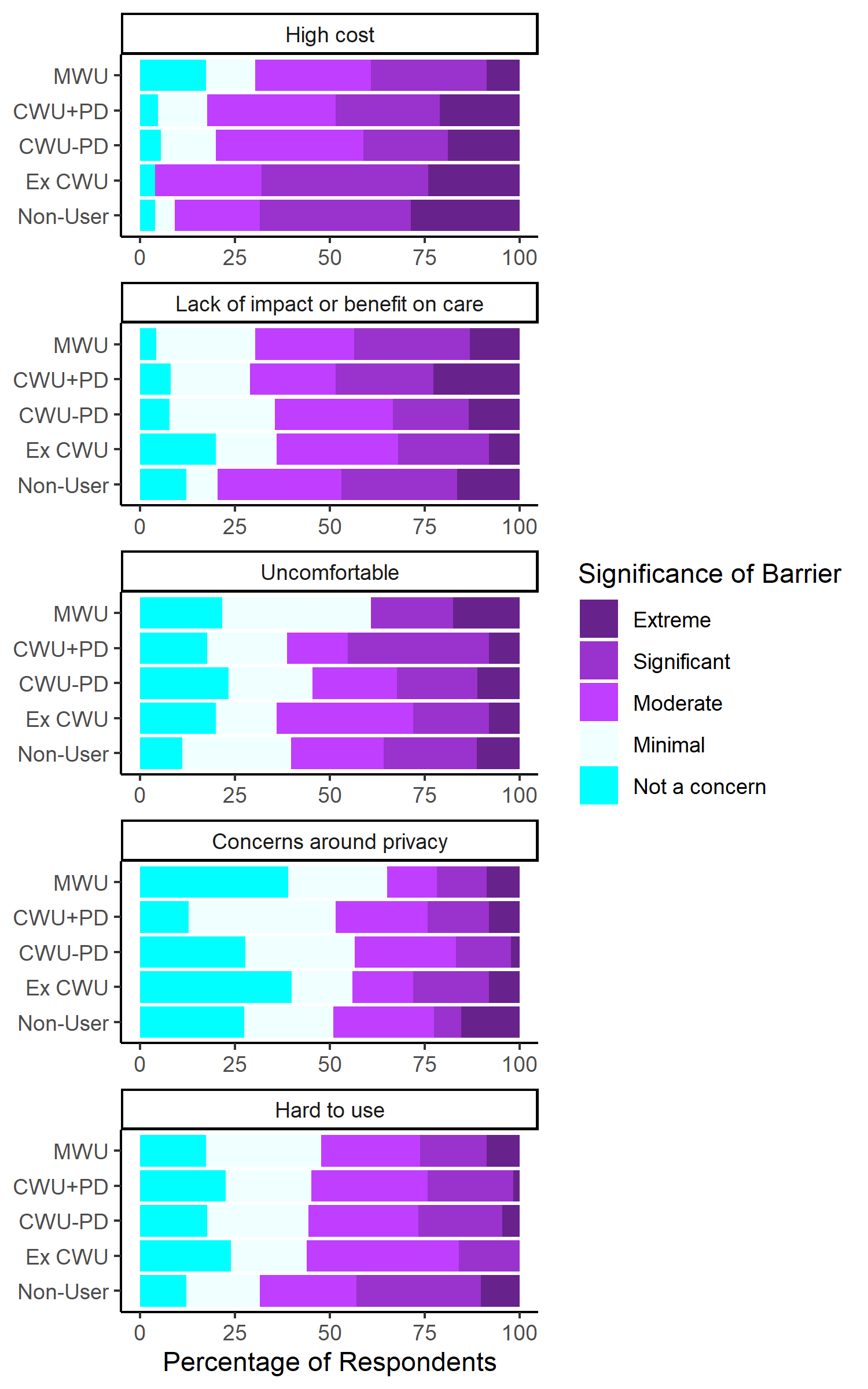

Supplement: Supplementary Figure S1 [file Image1.tiff]

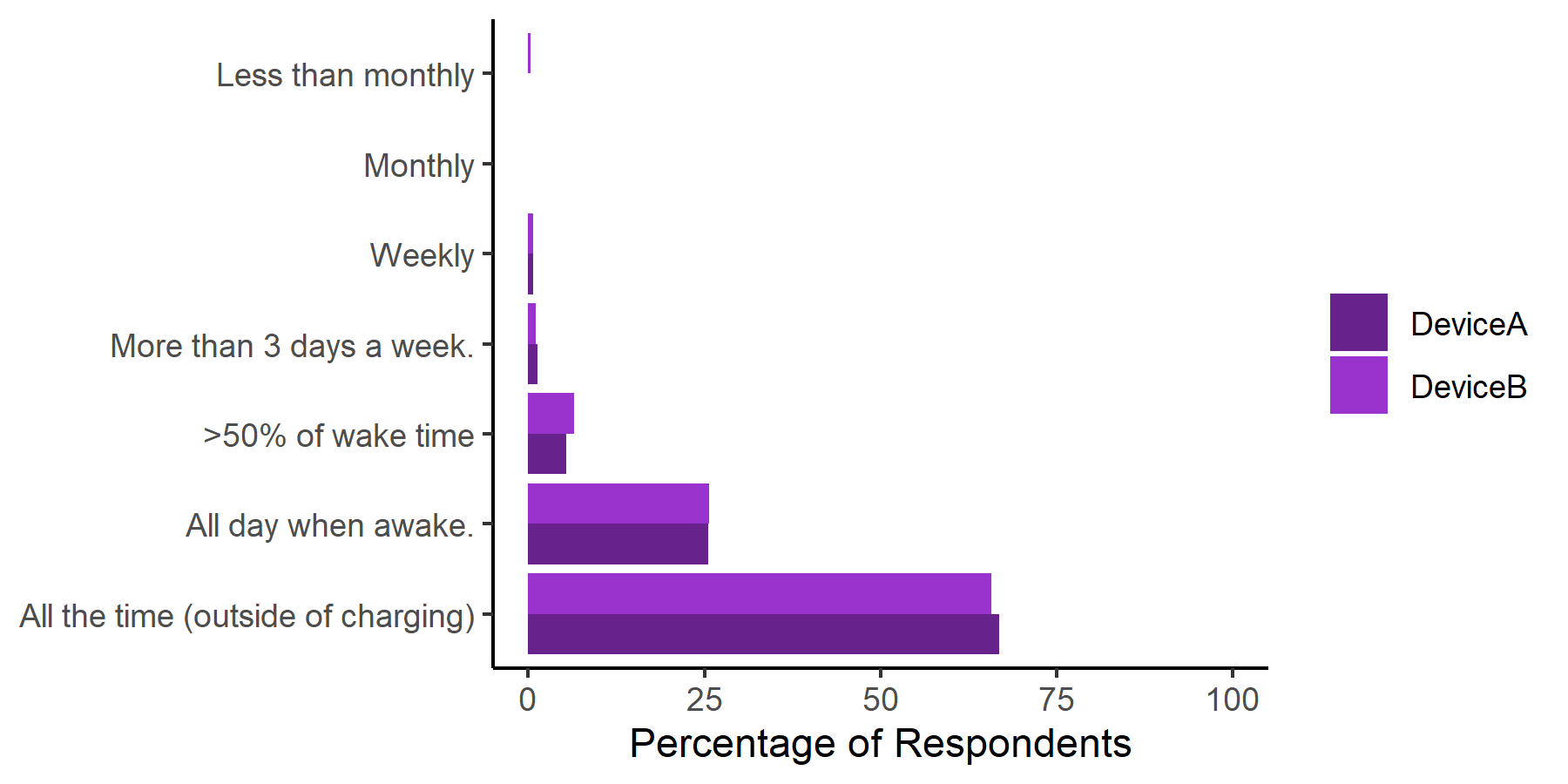

Supplement: Supplementary Figure S2 [file Image2.tiff]

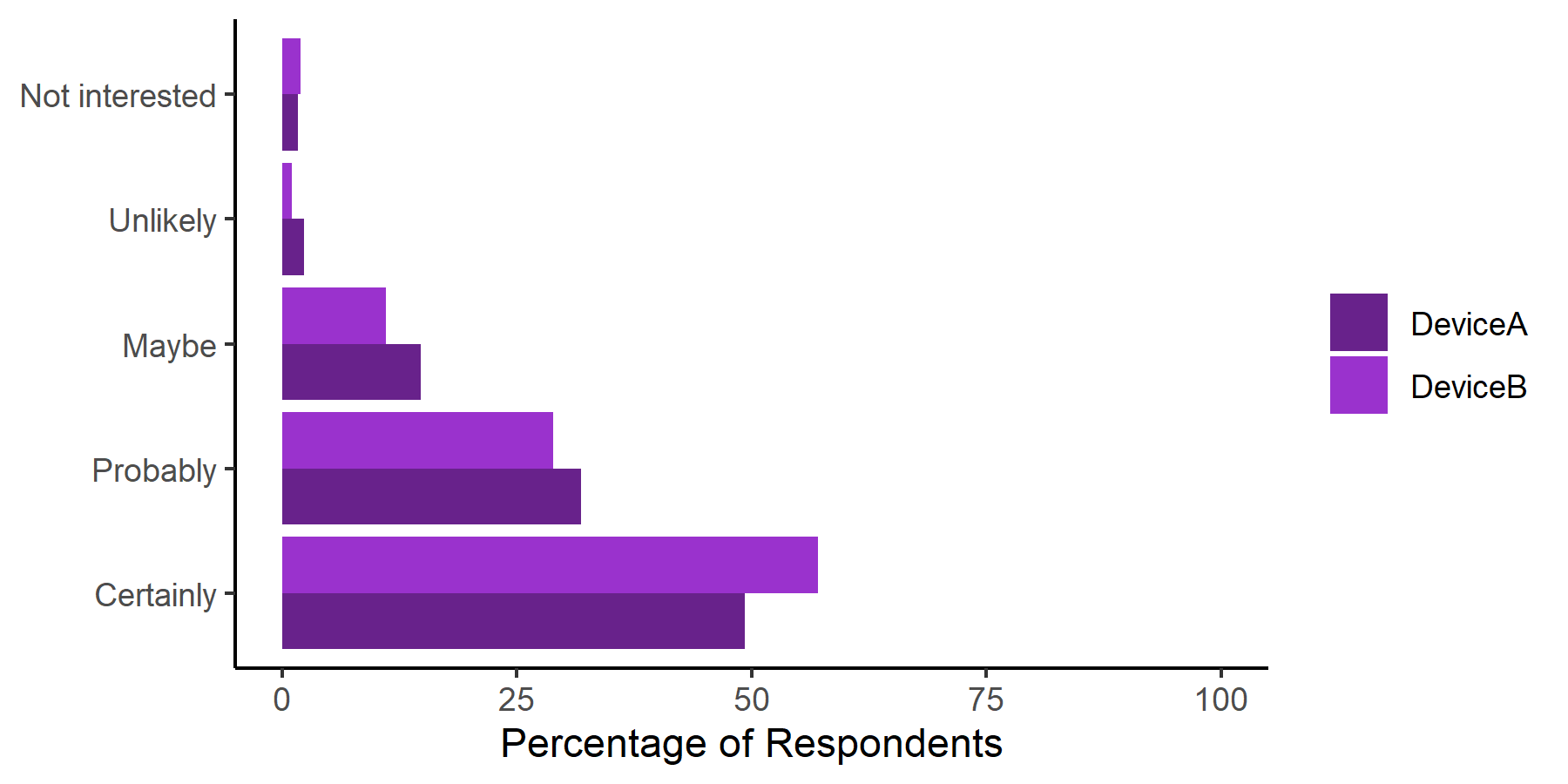

Supplement: Supplementary Figure S3 [file Image3.tiff]

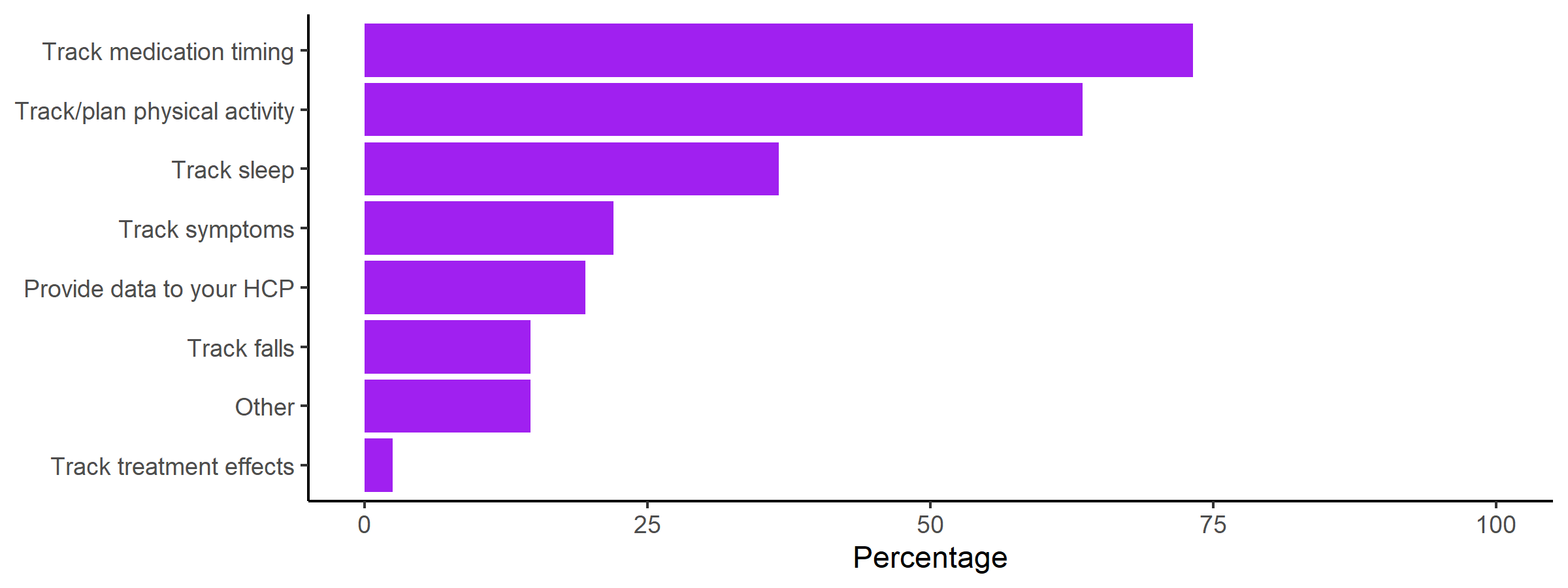

Supplement: Supplementary Figure S4 [file Image4.tiff]
