# Supplementary material for: The current state of wearable device use in Parkinson's disease: a survey of individuals with Parkinson's
Source: Front Digit Health. 2024 Dec 23;6:1472691. doi: 10.3389/fdgth.2024.1472691 (PMC11701158; doi:10.3389/fdgth.2024.1472691)
Supplement: Supplementary file 13 [file Datasheet1.pdf]

Confidential

Wearable Device Survey for Individuals with Parkinson's Disease

Survey Information

Thank you for your interest in this survey on wearable device use in Parkinson's Disease!

Purpose

We want to understand better what barriers limit the use of wearable devices in people with PD. By identifying and determining the significance of various barriers, we hope to then guide future solutions and further advance the care of people with PD.

Participants

This specific survey is for people diagnosed wth Parkinson's Disease who are over the age of 18 years. Caregivers and family members are welcome to assist with filling out the survey and/or with answering questions.

Procedure

This survey will be conducted anonymously and through this online tool (REDCap). If you consent to participating, then you will be asked on the following pages to answer questions about general demographics, basics on your PD, perceptions on technologies, and about your perceptions and experiences with wearable devices. Most questions will be multiple choice, but you are free to provide more feedback. The entire survey should take less than 15 minutes. Responses for most questions will be required, except for questions explicitly marked as optional. If you need to step away from the survey, you can do so and return at a later time.

If there are any questions please contact WearableSurveyPD@uw.edu and we will be happy to answer them.

Survey Consent

This is an anonymous survey which does not collect personally identifying information. The risk of the study are minimal. From a privacy perspective there is a very low chance of self-identification through answering questions. Questions are of a general factual nature or asking about opinions, as such there is low risk of emotional harm. However, this type of questioning can provoke negative emotions due to creating contrasts between ideals and realities. The direct benefits to participants of the survey are minimal and mostly related to increased awareness of the topic.

I consent to taking this survey.	◯ Yes<br>◯ No

Survey Definitions

Definitions

You - refers to you as an individual diagnosed with PD or, if indicated, as a caregiver of someone living with PD.

Wearable Device - any technological accessory which is affixed to the surface of an individual and which provides information on their movements. Typically, devices come in the form of smart watches or fitness trackers. For some questions, a paired device (smart phone) is considered part of the wearable system.

Example:

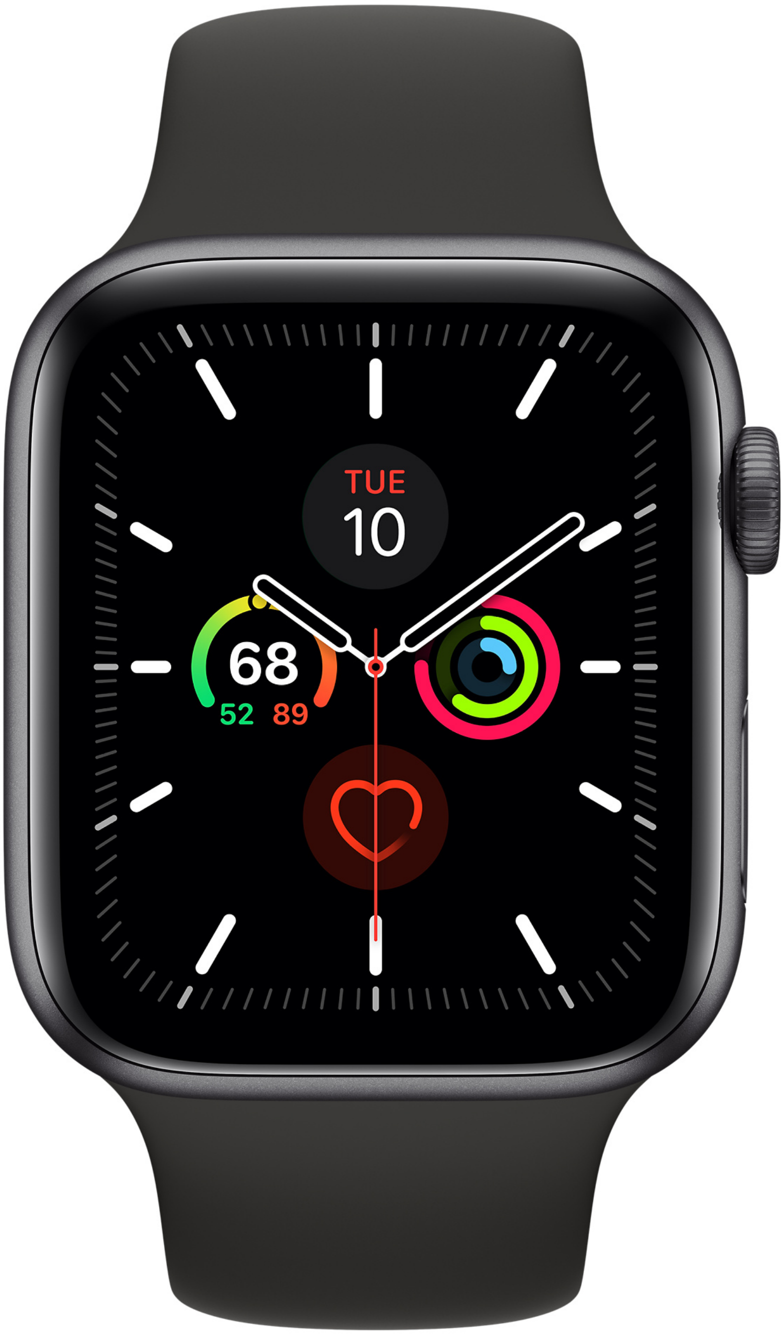

Basic Demographics

How old are you?

- ◯ < 40
- ◯ 40-50
- ◯ 50-60
- ◯ 60-70
- ◯ 70-80
- ◯ 80+

What gender do you identify as?

- ◯ Male
- ◯ Female
- ◯ Non-binary
- ◯ Prefer not to answer

What kind of health system do you get most of your Parkinson's disease care from?

- ◯ Tertiary/quaternary academic/university system
- ◯ Private or community non-university system system
- ◯ County/city public system
- ◯ Veteran's administration or other national governmental entity

What kind of area do you live in?

- ◯ Rural
- ◯ Suburban
- ◯ Urban

What state do you live in?	◯ AL<br>◯ AK<br>◯ AZ<br>◯ AR<br>◯ CA<br>◯ CO<br>◯ CT<br>◯ DC<br>◯ DE<br>◯ FL<br>◯ GA<br>◯ HI<br>◯ ID<br>◯ IL<br>◯ IN<br>◯ IA<br>◯ KS<br>◯ KY<br>◯ LA<br>◯ ME<br>◯ MD<br>◯ MA<br>◯ MI<br>◯ MN<br>◯ MS<br>◯ MO<br>◯ MT<br>◯ NE<br>◯ NV<br>◯ NH<br>◯ NJ<br>◯ NM<br>◯ NY<br>◯ NC<br>◯ ND<br>◯ OH<br>◯ OK<br>◯ OR<br>◯ PA<br>◯ RI<br>◯ SC<br>◯ SD<br>◯ TN<br>◯ TX<br>◯ UT<br>◯ VT<br>◯ VA<br>◯ WA<br>◯ WV<br>◯ WI<br>◯ WY<br>◯ AS<br>◯ GU<br>◯ MH<br>◯ PR<br>◯ UM<br>◯ VI
How interested are you in using new technologies?	◯ Very interested in new technologies<br>◯ Somewhat interested in new technologies<br>◯ Can go either way<br>◯ Somewhat disinterested in new technologies<br>◯ Very disinterested in new technologies

Basic Parkinson's Disease State Information

Which movement symptom of Parkinson's Disease did you first develop?	◯ Slowness<br>◯ Tremor<br>◯ Stiffness<br>◯ Imbalance<br>◯ Poor handwriting<br>◯ Other
Which other symptom did you develop?	__________________________________
Currently what symptoms of Parkinson's Disease affect your quality of life? (Select all that apply)	☐ Slowness<br>☐ Tremor<br>☐ Stiffness<br>☐ Dyskinesia<br>☐ Imbalance/Falls<br>☐ Insomnia<br>☐ Dizziness/Orthostasis<br>☐ Hallucinations
For how many years have you had movement related symptoms (tremor, slowness, changes in walking)?	◯ < 2<br>◯ 2-4<br>◯ 4-6<br>◯ 6-10<br>◯ >10
How often do you take medications in a day for PD?	◯ 1<br>◯ 2<br>◯ 3<br>◯ 4<br>◯ 5<br>◯ 6 or more
Do you use an assistive device to walk or get around?	◯ Yes<br>◯ No
Do you use a cane?	◯ No<br>◯ Rarely<br>◯ For long trips<br>◯ Sometimes<br>◯ Errands/when I go out<br>◯ Often<br>◯ Always
Do you use a walker?	◯ No<br>◯ Rarely<br>◯ For long trips<br>◯ Sometimes<br>◯ Errands/when I go out<br>◯ Often<br>◯ Always
Do you use a wheelchair?	◯ No<br>◯ Rarely<br>◯ For long trips<br>◯ Sometimes<br>◯ Errands/when I go out<br>◯ Often<br>◯ Always

Current Understanding and Use of Wearable Devices

Before being contacted about this survey had you heard about wearable devices?
- ◯ Yes
- ◯ No

If yes, which ones have you heard of? (Select all that apply)
- ☐ None in particular, just the concept
- ☐ Consumer smart watches devices (Apple Watch, Samsung, Google)
- ☐ Fitness trackers (Fitbit, Garmin, Oura)
- ☐ Medical devices
- ☐ Other

Which other wearable device have you heard of?

____

Do you currently use or have you ever used a consumer wearable device or wearable medical device?
- ◯ Yes
- ◯ No

Which device(s) have you used? (Select all that apply) (if you use the Apple Watch w/ StrivePD system, then please select both Apple Watch and Apple Watch w/ Strive PD)
- ☐ Apple Watch
- ☐ Apple Watch w/ StrivePD (Medical device)
- ☐ Android Watch
- ☐ Garmin
- ☐ Fitbit and other fitness trackers
- ☐ PKG (Medical device)
- ☐ KinesiaU (Medical device)
- ☐ PDMonitor (Medical device)
- ☐ Other

Which other device(s) have you used?

____

Consumer Wearable Device Use

If you are using a consumer wearable, which is your current preferred device?

- ◯ Not currently using a consumer wearable
- ◯ Apple Watch
- ◯ Android Watch
- ◯ Garmin
- ◯ Fitbit and other fitness trackers
- ◯ Other

Which other wearable device is your current preferred device?

____

How often do you use your current preferred consumer wearable device?

- ◯ Nearly always (outside of charging)
- ◯ All day when awake
- ◯ >50% of wake time
- ◯ Not daily but more than 3 days a week
- ◯ Weekly
- ◯ Monthly
- ◯ Less than monthly (or when treatment changes are made by my healthcare team or before visits with my healthcare team)
- ◯ No longer using

Do you or your caregiver use your preferred consumer wearable to help manage your PD?

- ◯ No
- ◯ No, but we did in the past with this device
- ◯ No, but we did in the past with another device
- ◯ Yes

How do you use your preferred consumer wearable to manage PD?

- ☐ Track and/or plan your physical activity.
- ☐ Track sleep.
- ☐ Track when medications are due or when take them.
- ☐ Track symptoms.
- ☐ Track falls or near falls.
- ☐ Track changes after your healthcare team changes medications or DBS settings
- ☐ Provide data to your healthcare team about how you are doing.
- ☐ Other reason (free response)

What other way are you using your preferred consumer wearable to manage your PD?

____

Do you or your caregiver feel that the data you get from the use of your preferred consumer wearable device impacts how you or your caregiver manage your symptoms?

- ◯ Substantially positively
- ◯ Somewhat positively
- ◯ Not particularly
- ◯ Somewhat negatively
- ◯ Substantially Negatively

Do you or your caregiver feel that the data you get from the use of your preferred consumer wearable device impacts how your healthcare team manages your symptoms?

◯ Substantially positively
◯ Somewhat positively
◯ Not particularly
◯ Somewhat negatively
◯ Substantially Negatively

If for the purpose of managing PD (e.g. scheduling medications, recording symptoms, tracking activity, etc.), you are not using a consumer wearable, or using one less than you would like what is limiting your use? (Check all that apply)

☐ Nothing. I am using a wearable (consumer or medical) and using it as much as I want to
☐ I didn't know that I could use it like that.
☐ I think it is uncomfortable or difficult to keep on.
☐ I think it requires too much effort to maintain.
☐ I can't track the data I want.
☐ It is too difficult or time consuming to input the data into the device.
☐ It is too difficult to review or access any of the data.
☐ I don't think the information is accurate.
☐ I don't think that the information helps me better understand or manage my Parkinson's.
☐ I don't think my health care provider will use the information.
☐ It costs too much
☐ Other:

What other factor is limiting your use of consumer wearables for the management of Parkinson's Disease? ________________________________

Medical Wearable Devices

How often do you use the Apple Watch with strivePD system?

- ◯ Nearly always (outside of charging)
- ◯ All day when awake
- ◯ >50% of wake time
- ◯ Not daily but more than 3 days a week
- ◯ Weekly
- ◯ Monthly
- ◯ Less than monthly (or when treatment changes are made by my healthcare team or before visits with my healthcare team)
- ◯ No longer using

How often do you the PKG?

- ◯ Nearly always (outside of charging)
- ◯ All day when awake
- ◯ >50% of wake time
- ◯ Not daily but more than 3 days a week
- ◯ Weekly
- ◯ Monthly
- ◯ Less than monthly (or when treatment changes are made by my healthcare team or before visits with my healthcare team)
- ◯ No longer using

How often do you use the kinesia system?

- ◯ Nearly always (outside of charging)
- ◯ All day when awake
- ◯ >50% of wake time
- ◯ Not daily but more than 3 days a week
- ◯ Weekly
- ◯ Monthly
- ◯ Less than monthly (or when treatment changes are made by my healthcare team or before visits with my healthcare team)
- ◯ No longer using

How often do you use the PDmonitor?

- ◯ Nearly always (outside of charging)
- ◯ All day when awake
- ◯ >50% of wake time
- ◯ Not daily but more than 3 days a week
- ◯ Weekly
- ◯ Monthly
- ◯ Less than monthly (or when treatment changes are made by my healthcare team or before visits with my healthcare team)
- ◯ No longer using

If for the purpose of managing PD, you are not using the Apple Watch with Strive PD system, or using it less than you would like what is limiting its use? (Check all that apply)

☐ Nothing. I am using it as much as I want to.
☐ It is uncomfortable or difficult to keep on.
☐ It requires too much effort to maintain.
☐ I can't input or track the data that I want.
☐ It is too difficult or time consuming to input the data into the device.
☐ It is too difficult to review or access any of the data.
☐ I don't think that the information helps me better understand or manage my Parkinson's.
☐ I don't think my health care provider uses the information.
☐ I don't think the information is accurate.
☐ My healthcare care provider discontinued it.
☐ It costs too much.
☐ Other:

What other factor is limiting the use of the Apple Watch with Strive PD?

____

If for the purpose of managing PD, you are not using the PKG, or using it less than you would like what is limiting its use? (Check all that apply)

☐ Nothing. I am using it as much as I want to.
☐ It is uncomfortable or difficult to keep on.
☐ It requires too much effort to maintain.
☐ I can't input or track the data that I want.
☐ It is too difficult or time consuming to input the data into the device.
☐ It is too difficult to review or access any of the data.
☐ I don't think that the information helps me better understand or manage my Parkinson's.
☐ I don't think my health care provider uses the information.
☐ I don't think the information is accurate.
☐ My healthcare care provider discontinued it.
☐ It costs too much.
☐ Other:

What other factor is limiting the use of the PKG?

____

If for the purpose of managing PD, you are not using the Kinesia, or using it less than you would like what is limiting its use? (Check all that apply)

☐ Nothing. I am using it as much as I want to.
☐ It is uncomfortable or difficult to keep on.
☐ It requires too much effort to maintain.
☐ I can't input or track the data that I want.
☐ It is too difficult or time consuming to input the data into the device.
☐ It is too difficult to review or access any of the data.
☐ I don't think that the information helps me better understand or manage my Parkinson's.
☐ I don't think my health care provider uses the information.
☐ I don't think the information is accurate.
☐ My healthcare care provider discontinued it.
☐ It costs too much.
☐ Other:

What other factor is limiting use of the Kinesia?

____

If for the purpose of managing PD, you are not using the PDmonitor, or using it less than you would like what is limiting its use? (Check all that apply)

- ☐ Nothing. I am using it as much as I want to.
- ☐ It is uncomfortable or difficult to keep on.
- ☐ It requires too much effort to maintain.
- ☐ I can't input or track the data that I want.
- ☐ It is too difficult or time consuming to input the data into the device.
- ☐ It is too difficult to review or access any of the data.
- ☐ I don't think that the information helps me better understand or manage my Parkinson's.
- ☐ I don't think my health care provider uses the information.
- ☐ I don't think the information is accurate.
- ☐ My healthcare care provider discontinued it.
- ☐ It costs too much.
- ☐ Other:

What other factor is limiting the use of the PDmonitor?

____

Overall do you or your caregiver feel that the data you get from medical wearable devices impacts how your healthcare team manages your symptoms?

- ○ Substantially positively
- ○ Somewhat positively
- ○ Not particularly
- ○ Somewhat negatively
- ○ Substantially Negatively

Overall do you or your caregiver feel that the data you get from medical wearable devices impacts how you or your caregiver manages your symptoms?

- ○ Substantially positively
- ○ Somewhat positively
- ○ Not particularly
- ○ Somewhat negatively
- ○ Substantially Negatively

Application Use

Do you use any smartphone or smartwatch applications for the monitoring of your Parkinson's disease?

◯ Yes ◯ No

Which apps do you use?

Theoretical Devices:

Would you use a theoretical wearable system that helps you better understand your disease and responses to medical interventions, but it doesn't directly provide information to your healthcare team?

◯ Certainly
◯ Probably
◯ Maybe
◯ Unlikely
◯ Not interested

How much time during the day would you be willing to have this type of device on you? (Assume no issues with comfort and charging)

◯ All the time (outside of charging)
◯ All day when awake.
◯ >50% of wake time
◯ More than 3 days a week.
◯ Weekly
◯ Monthly
◯ Less than monthly or when treatment changes are made by my healthcare team or before visits with my healthcare team.

Would you be willing to pay a fee to have such a device?

◯ No, insurance must cover it.
◯ Yes

If you are willing to buy the device, what is the maximum that you would be willing to pay (in USD).

____

If you are willing to pay for the device, how much would the most you would be willing to pay for a monthly subscription (in USD/month)?

____

Theoretical Devices:

Would you use a theoretical wearable system that helps you understand your disease better and helps your healthcare team manage your medications and/or electrical stimulation by providing data directly to them for review?

◯ Certainly
◯ Probably
◯ Maybe
◯ Unlikely
◯ Not interested

How much time during the day would you be willing to have this type of device on you? (Assume no issues with comfort and charging)

◯ All the time (outside of charging)
◯ All day when awake.
◯ >50% of wake time
◯ More than 3 days a week.
◯ Weekly
◯ Monthly
◯ Less than monthly or when treatment changes are made by my healthcare team or before visits with my healthcare team.

If you are willing to buy the device, what is the maximum that you would be willing to pay (in USD). __________________________________

If you are willing to pay for the device, how much would the most you would be willing to pay for a monthly subscription (in USD/month)? __________________________________

Many new technologies have limitations and individuals can have concerns with their use. For the following concerns please indicate whether they are ones you share and to what degree they affect your use of wearables for PD.

	Extreme	Significant	Moderate	Minimal	Not a concern
Hard to use	◯	◯	◯	◯	◯
Uncomfortable	◯	◯	◯	◯	◯
Concerns around privacy	◯	◯	◯	◯	◯
High cost	◯	◯	◯	◯	◯
Lack of impact or benefit on care	◯	◯	◯	◯	◯
Other	◯	◯	◯	◯	◯

If you have another barrier to use of wearables, please let us know what it is.

____

If you would like to provide any other commentary about the use of wearable devices in the management of Parkinson's disease. We would love to hear from you. Feel free to write below.

As a security measure you are welcome to input a unique 10 or more number, digit, and/or symbol "key". If you hold on to this key and the time of your survey completion, if any significant issues with survey collection occur this will allow us to establish the authenticity of your answers while allowing continued anonymity.

____
